# The relationship between linguistic expression in blog content and symptoms of depression, anxiety, and suicidal thoughts: A longitudinal study

**DOI:** 10.1371/journal.pone.0251787

**Published:** 2021-05-19

**Authors:** Bridianne O’Dea, Tjeerd W. Boonstra, Mark E. Larsen, Thin Nguyen, Svetha Venkatesh, Helen Christensen

**Affiliations:** 1 Faculty of Medicine, Black Dog Institute, University of New South Wales, Sydney, New South Wales, Australia; 2 Applied Artificial Intelligence Institute, Deakin University, Burwood, Victoria, Australia; Lancaster University, UNITED KINGDOM

## Abstract

Data generated within social media platforms may present a new way to identify individuals who are experiencing mental illness. This study aimed to investigate the associations between linguistic features in individuals’ blog data and their symptoms of depression, generalised anxiety, and suicidal ideation. Individuals who blogged were invited to participate in a longitudinal study in which they completed fortnightly symptom scales for depression and anxiety (PHQ-9, GAD-7) for a period of 36 weeks. Blog data published in the same period was also collected, and linguistic features were analysed using the LIWC tool. Bivariate and multivariate analyses were performed to investigate the correlations between the linguistic features and symptoms between subjects. Multivariate regression models were used to predict longitudinal changes in symptoms within subjects. A total of 153 participants consented to the study. The final sample consisted of the 38 participants who completed the required number of symptom scales and generated blog data during the study period. Between-subject analysis revealed that the linguistic features “tentativeness” and “non-fluencies” were significantly correlated with symptoms of depression and anxiety, but not suicidal thoughts. Within-subject analysis showed no robust correlations between linguistic features and changes in symptoms. The findings may provide evidence of a relationship between some linguistic features in social media data and mental health; however, the study was limited by missing data and other important considerations. The findings also suggest that linguistic features observed at the group level may not generalise to, or be useful for, detecting individual symptom change over time.

## Introduction

Worldwide, depression and anxiety are leading causes of disability and represent a major health and economic burden [1]. This is due in part to the detrimental effects of these mental illnesses on functioning, but also the low levels of mental health literacy among individuals, the inability to recognise symptoms, poor help-seeking attitudes, and lack of access to care [2–4]. A fatal and tragic outcome of poor mental health is suicide, a primary cause of death for both young and middle-aged people worldwide [5]. There is a need to look to new ways of detecting mental illness in the population, particularly in the prodromal phase, to increase treatment outcomes, reduce severity, and prevent death [6]. Social media has emerged as a potential means for doing this [7].

Defined as any internet-enabled platform that allows individuals to connect, communicate, and share content with others, social media platforms include social networking sites (e.g. Facebook), microblogs (e.g. Twitter), blog sites (e.g. WordPress, LiveJournal), and online forums (e.g. Reddit) [8]. There has been significant enthusiasm in the potential of these platforms to generate markers of mental health as they are used by millions of people worldwide, data is produced in natural settings, is readily available and at no cost. It has been hypothesised that the language and expressive features within individuals’ shared social media content may indicate their mental state [9]. This is based on psycho-linguistic theory which postulates that the words and features used in everyday language can reveal individuals’ thoughts, emotions, and motivations [10–12]. Several promising findings have emerged.

On Twitter, De Choudhury et al [13] was able to discriminate depression among users by the increased use of first person pronouns and fewer references to third persons. Statistical modelling was most accurate when only linguistic features were used. Using cross-validation methods, Reece et al [14] found that depression among Twitter users was predicted by differences in word count, references to ingestion, sadness, swear words, article words, and positive emotion. Also on Twitter, Tsugawa et al [15] found depressed users had significantly higher ratios of negative emotion words. Wilson et al [16] found Twitter posts with depression terms were characterised by higher character counts, fewer pronouns, less positive emotion, greater negative emotion, greater expressions of sadness, fewer references to time and fewer references to past and present tense. When comparing posts in online depression forums with those in breast cancer forums, Ramirez-Esparza et al [17] found posts in depression forums were characterised by greater first-person referencing, less positive emotion, and greater negative emotion.

When comparing Facebook and Twitter, Seabrook et al [18] found depression on Facebook was characterised by differences in the proportion of negative emotions whereas depression on Twitter was associated with less dispersion of negative emotion across posts. Also on Facebook, Eichstaedt et al [19] found depression to be marked by increased first-person pronoun use, greater negative emotion, increased perceptual processes for feeling (e.g. feels, touch), greater references to sadness (e.g. crying, grief), and greater discrepancies (e.g. should, would, could). Our team [20] discriminated higher risk in suicide-related Twitter posts by greater self-references, anger, and a focus on the present [9]. These findings are generally consistent with a recent meta-analysis that confirmed the increased use of first person singular pronouns is a universal linguistic marker of depression [21]. Taken together, past studies support the potential of utilising social media content for the automatic detection of mental health problems; although, the field remains hampered by major methodological challenges.

A major limitation of past studies is the lack of validation between the various linguistic features and psychometric measures of mental health. A review of recent papers in this field [22] found that of the 12 included studies, only five used valid mental health questionnaires [13–15,23,24]. The remaining relied on self-declared diagnoses (e.g., affirmative statements of mental health diagnoses in social media posts), membership associations (e.g. belonging to a certain online community or forum), or annotation of content (e.g. presence of key words or phrases). As such, it is not clear whether all past findings are consistent with diagnostic criteria. Further, most of the past studies in this area have focussed only on depression. Although this is warranted, due to the prevalence and associated costs, little attention has been paid to other mental health symptoms such as anxiety or suicidality, which are highly correlated with depression and equivalent in distress and disability [25]. Detecting these mental health problems may be an effective way to identify those who have depression or who are at risk of developing it [26].

Based on past studies, it also remains unclear whether group-level markers derived from social media data can be used to infer the mental health state of individual users. Current knowledge is mostly based on cross-sectional studies, due to the administrative and participant burdens associated with longitudinal research. As a result, the temporal patterns in symptomatology have remained largely unaccounted for [27]. Previous research in psychological science has shown that relationships observed at the group level do not necessarily generalise to all individuals within a sample [28–30]. Indeed, the field of personalised medicine argues that individuals have unique markers of mental ill-health [31]. We should hence carefully examine, rather than assume, whether relationships observed at the group level in past studies also hold for individuals over time [30]. Intensive repeated-measures data, in larger samples, are needed to make predictions about intra-individual changes in mental health scores over time.

### Study objectives

The current study aimed to overcome some of these past limitations by collecting validated mental health data in a longitudinal study of individuals who blog. Web blogs are a social media platform that allow individuals to publish a chronological series of discrete, often informal diary-style text entries that convey their thoughts, feelings, and attitudes, to a community of followers. These followers can interact with this content by leaving comments, “likes”, and contributing to the total “views”. Blog sites may offer an ideal platform for the identification of linguistic markers of mental health due to the abundance of text data, the sequential nature of content, and the anonymity that many blog sites provide. The current study explored the relationship between linguistic features and symptoms of depression, anxiety, and suicidal ideation using the text content extracted from individuals’ blog sites. Guided by past studies, it was hypothesised that at the group-level, higher mental health symptom scores would be associated with increased references to oneself, increased expressions of negative emotion, and reduced references to third persons. This study also examined whether the group-level correlations could be used to make predictions about intra-individual changes in mental health scores over time. These outcomes may help to establish prediction models which can be used to monitor blog sites automatically, and in real time, for mental health risk.

## Method

### Study design

A 36-week prospective longitudinal cohort study with mental health data and blog content collected fortnightly.

### Ethics statement

The study was approved by University of New South Wales (14086) and Deakin University (2014187) Human Research Ethics Committee. All data was collected and used with explicit consent from participants and according to the Terms and Conditions of the social media platforms at the time of the study. To protect participants’ privacy, no identifiable or raw text data is published by the research team.

### Participants, recruitment, and consent

Recruitment took place between July 2014 and October 2016. Using a series of online adverts published on various social media platforms (Facebook, Twitter, Instagram, LiveJournal) and the Black Dog Institute website, individuals who blogged were invited to visit the study website where they were provided with the online Participant Information and Consent Form (PICF). Participants were required to confirm their age (above 18 years old), access to the Internet, use of a valid email address that does not contain their full name, and provide the URL of their blog site. Upon providing consent, participants undertook an online self-report mental health assessment at baseline, and then every fortnight via email for a period of 36 weeks (total of 18 assessments). These assessments could be completed on any Internet-enabled device. Support contacts were provided to the entire sample and there were no restrictions on help-seeking behaviour throughout the study. Participants received a reimbursement of 20AUD in Amazon webstore credit if they remained in the study at 16 weeks and then an additional reimbursement if they remained at 36 weeks (maximum reimbursement received was 40AUD).

### Measures

Demographics were assessed using questions on age, gender, prior diagnosis of depression or anxiety from a medical practitioner, and medication use for depression and anxiety. Participants were asked to rate their overall health as very bad, bad, moderate, good, very good. Depressive symptoms were assessed using the validated, self-report Patient Health Questionnaire (PHQ-9) [32]. This nine-item questionnaire assessed the presence of depressive symptoms in the past two weeks. Individuals were asked to rate the frequency of depressive symptoms using a four-point Likert scale ranging from “none of the time” to “every day or almost every day”. A total score is then calculated which can be classified as “nil-minimal” (0–4), “mild” (5–9), “moderate” (10–14), “moderately severe” (15–19) or “severe” (20+). Anxiety symptoms were assessed using the validated, self-report Generalised Anxiety Disorder Scale (GAD-7) [33]. This seven-item questionnaire assessed the presence of generalised anxiety symptoms in the past two weeks. It used the same response scale as the PHQ-9 and participants’ total scores can also be classified into the same severity categories. Participants were also asked if they had had a panic attack in the past two weeks, and if so, how many. Suicidal thoughts scores were based on participants’ responses to item 9 of the PHQ-9 (ranges from 0 to 3). Participants who reported that they experienced “thoughts that they would be better off dead, of harming themselves” for more than several days (i.e., score > 0) were deemed to have suicidal thoughts.

### Blog data extraction and linguistic analysis

Blog data was extracted fortnightly using the publicly accessible Application Programming Interface (APIs) for each platform, including Tumblr, Live Journal, WordPress, and BlogSpot. Blog posts were analysed using the Linguistic Inquiry and Word Count (LIWC) tool for linguistic features [34]. This software analysed the blogs posts and calculated the percentage of words that reflected the different emotions, thinking styles, social concerns and parts of speech captured by the LIWC program dictionary [35]. This resulted in a set of 68 linguistic features for each post. The tool also calculated the total number of words within the posts that match the program dictionary, reported as “dictionary words”. LIWC scores were averaged for the participants who made more than one blog post during the two-week assessment period. This resulted in a dataset consisting of participants’ symptom scores matched with their averaged LIWC scores for the same period (see data repository file titled *groundtruth_individualdata*).

### Statistical analysis

We first performed bivariate analyses to investigate the correlation between the linguistic features and symptoms scores between individuals. To this end, we averaged the linguistic features scores and symptom scores across the assessment time points for each participant (see data repository file titled *groundtruth*_meandata). We performed bivariate analysis between the 68 linguistic features and the three symptom scores (depression, anxiety, suicidal ideation) using Spearman’s rank-order correlation. Hence, a total of 68 × 3 = 204 comparisons were performed. We used permutation tests to control the family-wise error rate [36,37]. A permutation was constructed by exchanging the symptom scores across participants and a new correlation coefficient was recomputed for the permuted data. This process was repeated for 10,000 permutations, resulting in the distribution of possible correlations coefficients for these data under the null hypothesis that observations are exchangeable. This procedure can be generalised to a family of similar tests by computing the distribution of the most extreme statistic (here the most extreme positive or negative correlations coefficient) across the entire family of tests for each permutation. This procedure corrects for multiple comparisons because the distribution of extreme statistics, from which the p-values of each comparison are derived, automatically adjusts to reflect the increased chance of false discoveries due to an increased number of comparisons [37]. We used the Matlab function *mult_comp_perm_corr*.*m* (https://au.mathworks.com/matlabcentral/fileexchange/34920-mult-comp-perm-corr) to perform the mass bivariate analyses. We used bootstrapping to estimate the confidence interval for the correlation coefficients [38].

#### Multivariate regression

We then performed multivariate analysis between multiple linguistic features using partial-least squares (PLS) regression. PLS regression is a multivariate extension of linear regression that builds prediction functions based on components extracted from the co-variance structure between features and targets [39]. Although it is possible to calculate as many PLS components as the rank of the target matrix, not all of them are normally used as data are never noise-free and extracting too many components will result in overfitting. Both the features and targets were z-transformed before performing PLS regression analysis. We used 5-fold cross-validation [40] to determine the number of components of the model and prediction accuracy was assessed using Mean Square Error (MSE). To select the best model, the number of components was increased until the MSE not further decreased. We used the built-in Matlab function *plsregress*.*m* from the Statistics and Machine Learning Toolbox (R2018a) to perform PLS regression. We performed PLS regression both on the full and a restricted feature set. To obtain a restricted feature set, we used bootstrapping: The z-scores of the feature loadings were estimated across 10,000 bootstrap samples and the four features with the largest z-score were selected. The PLS regression procedure was then repeated using only these four features.

#### Within-subject prediction

The previous analyses constructed regression models that predicted the symptom scores of participants that were not included in the training set. These group-level inferences do not necessarily generalise to intra-individual changes in symptom scores over time [28]. We therefore tested the PLS regression model constructed on group-level data on repeated measures of single participants using a two-staged approach. We first used the group-level model to predict the symptom scores at each time point at which linguistic features were extracted and symptoms were assessed and correlated the predicted and observed symptom scores across time points for each participant. We then compared the correlation coefficients estimated for each participant at the group level. To do this, we converted the correlation coefficients using Fisher’s z transformation and compared the z-scores against zero using a one-sample t-test.

## Results

### Participants

A total of 153 individuals consented to the study and completed the baseline assessment (88% female, mean age: 29.5 years, SD: 10.3, age range: 18–67, see S1 for more information). The final sample reported here consisted of the 38 participants who completed at least one mental health assessment and generated blog data for the same period. Table 1 outlines participant characteristics at baseline.

**Table 1:** Participant characteristics at baseline (N = 38).

	**M (SD)**	**Range**
Age	27.1 (7.2)	18–59
PHQ-9 score	14.5 (5.6)	3–27
GAD-7 score	11.6 (5.0)	0–20
Suicide/Self-harm score	1.07 (1.1)	0–3
Number of panic attacks experienced	7.0 (11.1)	0–60
	**N**	**%**
Female	32	84
Self-rated health		
Very bad	0	0
Bad	11	29
Moderate	15	39
Good	12	32
Very good	0	0
Prior mental health diagnosis by medical practitioner	36	95
Currently taking medication for mental health	26	68
Currently experiencing suicidal thoughts	25	66
Experienced a panic attack in 2 weeks prior	22	58
Depression symptom severity (PHQ-9)		
Nil-minimal	2	5
Mild	6	16
Moderate	10	26
Moderately-severe	11	29
Severe	9	24
Generalised Anxiety symptom severity (GAD-7)		
Nil-minimal	3	8
Mild	9	24
Moderate	15	39
Moderately-severe	9	24
Severe	2	5
Blog site used		
LiveJournal	30	79
Tumblr	8	21

**Fig 1:**
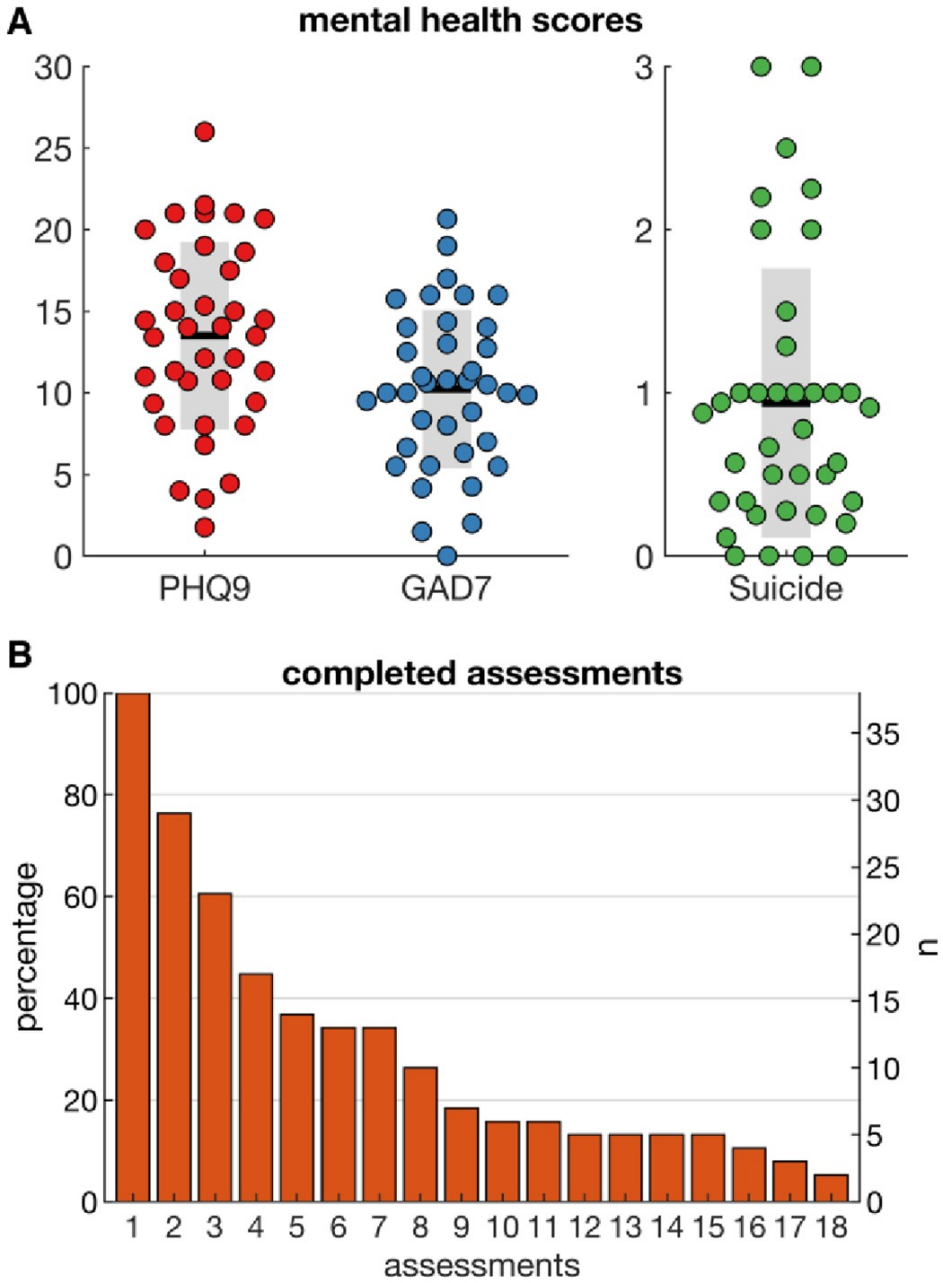
Characteristics of sample. A) Mental health scores (PHQ-9, GAD-7 and suicidal ideation) averaged across assessments and B) Number of mental health assessments completed by participants. Coloured dots show values of individual participants, the horizontal black line the group mean and the grey bars the SD.

On average, participants had moderately severe levels of depression and anxiety throughout the study assessments, but symptoms varied considerably between participants (PHQ-9 SD: 5.7, range: 1.8–26.0; GAD-7 SD: 4.8, range: 0–20.7; Fig 1A). Intra-individual differences between mental health scores showed a much smaller spread (S1 Fig in S1 File): the mean of the intra-individual standard deviation was 2.8 (SD: 2.7, range: 0–10.1) for the PHQ-9 and 2.2 (SD: 2.0, range: 0–7.6) for the GAD-7. Fig 1 shows the number of mental health assessments completed across the study period, with only 2 participants completing all 18. In the final sample, participants completed an average of 7.55 (SD:5.50) of the PHQ-9 scales, 7.26 (SD:5.47) of the GAD-7 scales.

Throughout the study period, there was also significant variability in the frequency of blog posts and word counts among participants (see Table 2). On average, participants posted blog content 32.92 times (SD: 58.41, range: 1 to 329), with an average of 192.76 (SD:170.52) words per blog post and a total mean word count of 3871.21 (SD: 5402.87, range 6 to 26947).

**Table 2:** Total number of surveys completed, blog posts, and word counts.

Participant Identification code	PHQ-9s completed	GAD-7s completed	Total posts	Total word count	Mean word count per post
1	9	9	6	2695	449.17
2	1	1	23	11004	478.43
3	3	3	4	2227	556.75
4	9	9	32	1822	56.94
5	1	1	4	1009	252.25
6	1	1	2	376	188.00
7	7	7	91	26947	296.12
8	3	3	5	922	184.40
9	9	9	13	2612	200.92
10	9	7	47	6773	144.11
11	9	9	4	506	126.50
12	17	17	8	3875	484.38
13	9	9	34	7686	226.06
14	9	9	21	3041	144.81
15	18	18	35	3909	111.69
16	1	1	1	217	217.00
17	9	9	1	6	6.00
18	10	10	5	1426	285.20
19	9	9	4	597	149.25
20	5	5	11	1102	100.18
21	6	6	39	4981	127.72
22	1	1	1	316	316.00
23	9	8	6	555	92.50
24	18	18	34	5585	164.26
25	6	1	6	1417	236.17
26	18	18	55	11804	214.62
27	18	18	128	15718	122.80
28	1	1	2	1564	782.00
29	17	14	3	58	19.33
30	8	8	3	318	106.00
31	4	4	115	3729	32.43
32	9	9	37	9481	256.24
33	1	1	18	1100	61.11
34	1	1	1	18	18.00
35	9	9	54	1564	28.96
36	2	2	61	1266	20.75
37	9	9	329	8545	25.97
38	2	2	8	335	41.88

### Between-subjects analysis

**Fig 2:**
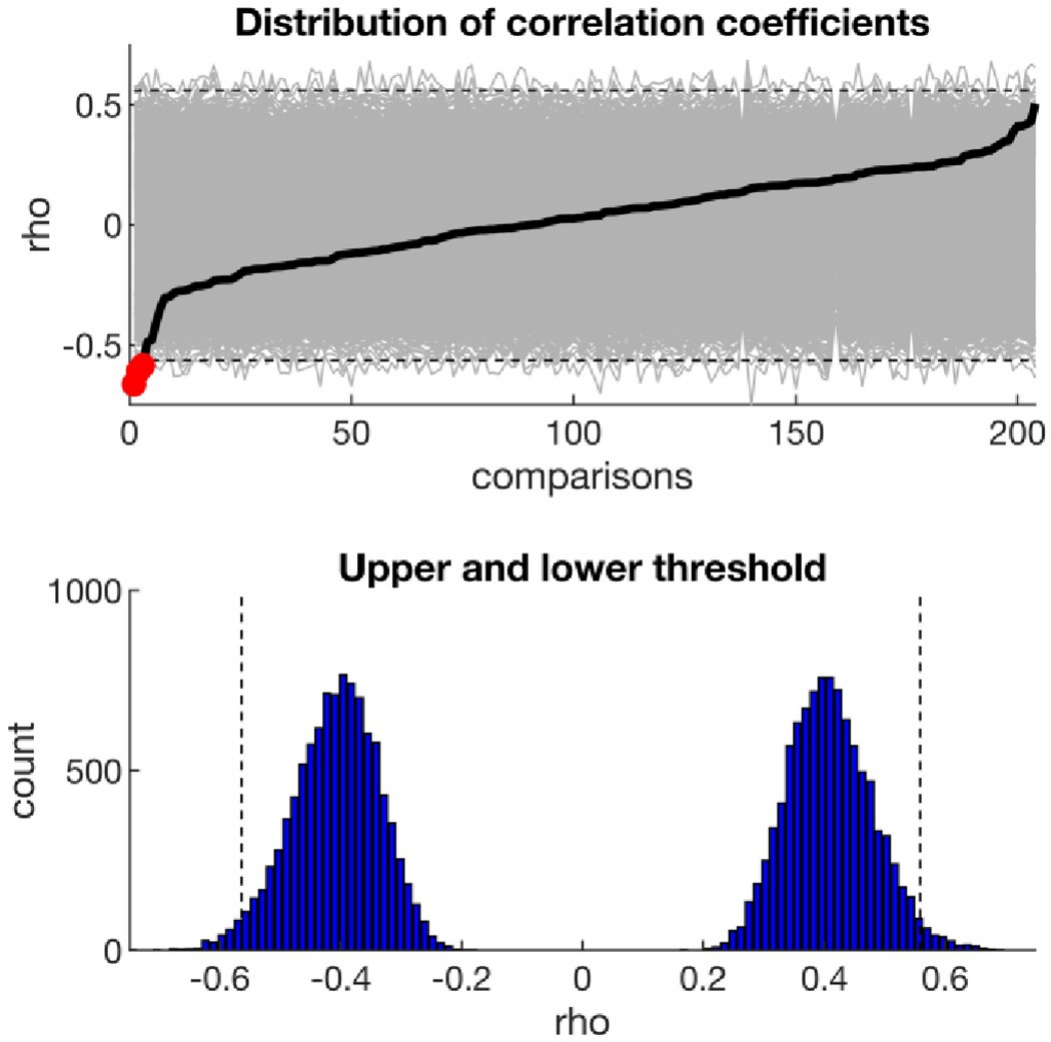
Permutation testing to control the family-wise error rate of mass bivariate analyses. Top panel shows the correlation coefficients for all 204 comparisons (black line) as well as the correlation coefficients of the 10,000 permutations (grey lines). The distribution of the most extreme statistic (bottom panel) was then used to determine adjusted significance threshold (dash line). Using this threshold only a few correlations are statistically significant (red dots, top panel).

We first performed mass bivariate analyses correlating the linguistic features with the three symptom scores. We used permutation tests to compute the distribution of the most extreme statistic across all comparisons and control the family-wise error rate. The resulting significance threshold was |*rho*| > 0.56 (Fig 2).

Table 3 outlines the linguistic features which showed the strongest correlations with depression, anxiety, and suicidal thoughts, respectively. After controlling for multiple comparisons, only non-fluencies were significantly correlated with depression (rho = -0.61, 95%CI: -0.77, -0.39, *P*_*corr*_ = 0.012), and non-fluencies and tentativeness with anxiety (rho = -0.58, 95%CI: -0.75, -0.31, *P*_*corr*_ = 0.028 and rho = -0.67, 95%CI: -0.79, -0.47, *P*_*corr*_ = 0.002, respectively).

**Table 3:** Between-subject analysis.

target	feature	example	rho	95% CI	*P*_uncorr_	*P*_corr_
depression	present focus	*is*, *does*, *here*	0.42	0.14, 0.62	0.008	0.84
	sadness	*crying*, *grief*, *sad*	0.41	0.12, 0.63	0.010	0.93
	certainty	*always*, *never*	0.39	0.08, 0.63	0.016	1
	3^rd^ person plural	*they*, *their*, *they’d*	-0.34	-0.59, -0.04	0.034	1
	tentative	*maybe*, *perhaps*,	-0.49	-0.66, -0.23	0.002	0.28
	non-fluencies	*er*, *hm*, *umm*	-0.61	-0.77, -0.39	0.000	**0.012**
anxiety	present focus	*is*, *does*, *here*	0.50	0.21, 0.71	0.001	0.21
	First person singular	*I*, *me*, *mine*	0.35	0.06, 0.60	0.031	1
	total pronouns	*I*, *them*, *itself*	0.30	0, 0.56	0.068	1
	future focus	*will*, *gonna*	-0.41	-0.66, -0.08	0.011	0.96
	tentative	*maybe*, *perhaps*	-0.58	-0.75, -0.31	0.000	**0.028**
	non-fluencies	*er*, *hm*, *umm*	-0.67	-0.79, -0.47	0.000	**0.002**
suicidality	ingesting	*dish*, *eat*, *pizza*	0.43	0.13, 0.66	0.007	0.74
	impersonal pronouns	*it*, *it’s*, *those*	0.41	0.09, 0.69	0.010	0.94
	certainty	*always*, *never*	0.32	0, 0.58	0.047	1
	relativity	*area*, *exist*, *stop*	-0.28	-0.53, 0.02	0.093	1
	space	*down*, *in*, *thin*	-0.28	-0.54, 0.01	0.084	1
	non-fluencies	*er*, *hm*, *umm*	-0.48	-0.69, -0.20	0.002	0.32

**Fig 3:**
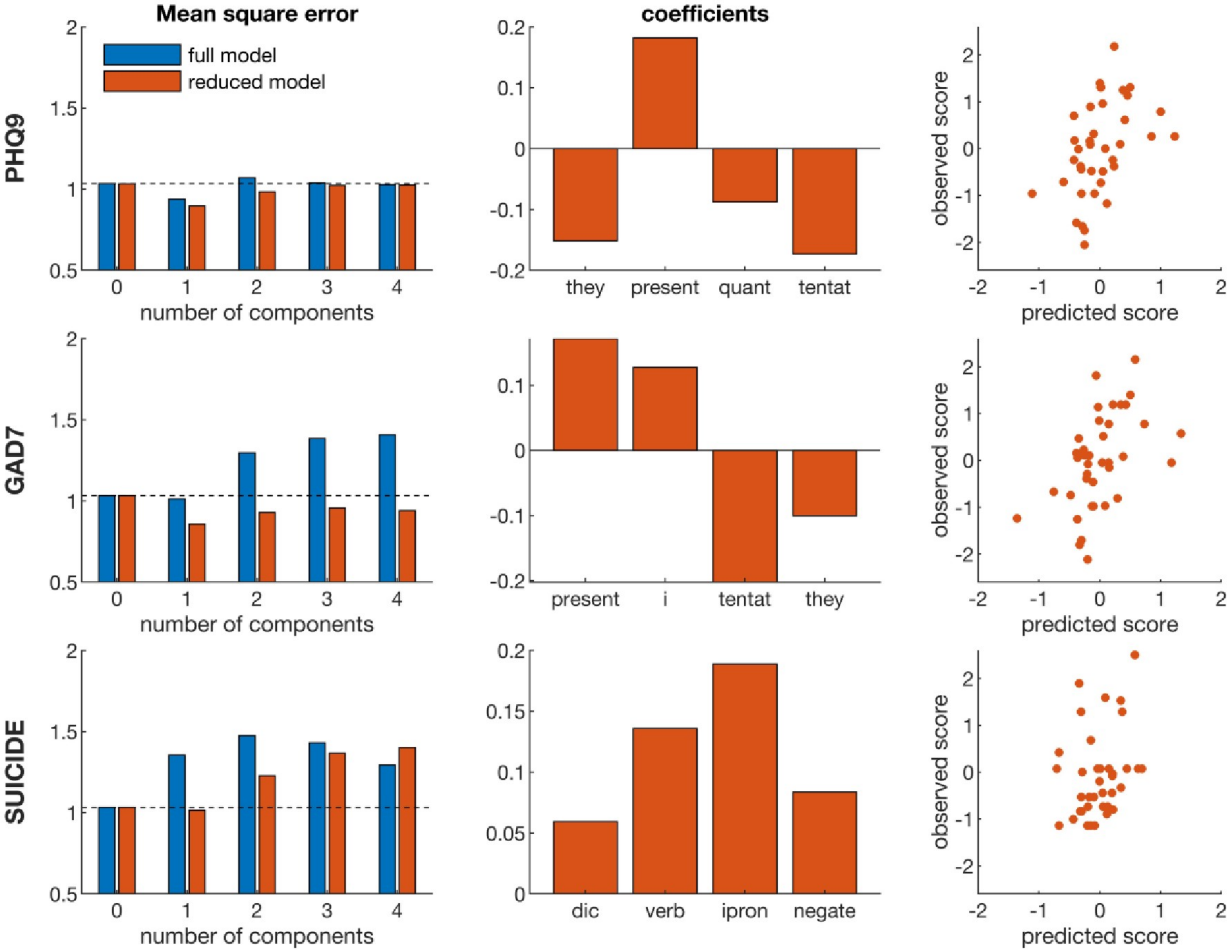
Results of PLS regression at group level. We used 5-fold cross-validation to determine the number of PLS components. The optimal model was the model showing the lowest MSE (left panel). We tested both the full model using all 68 linguistic features and a reduced model using only the 4 most robust features. The beta coefficients of the optimal model (middle column) were then used to estimate the predicted mental health scores (right column). Note: they = 3^rd^ person plural, present = present focus, quant = quantifiers, tentat = tentative, i = first person pronouns, dic = dictionary words, verb = auxiallary verbs, ipron = impersonal pronouns, and negate = negative emotion.

We then performed PLS regression to extract multiple linguistic features that predicted mental health scores. We first used all 68 linguistic features and used 5-fold cross-validation to determine the optimal number of components. For PHQ-9 and GAD-7, a PLS model with one component revealed a reduction in MSE (-9% and -2%, respectively), while no PLS model showed a reduction in MSE compared to a model with zero components for suicidal thought (Fig 3, left column). We used bootstrapping to determine the features that were most robust across participants. The four features with the highest absolute z-scores were ‘3^rd^ person pronouns’, ‘present focus’, ‘quantifiers’ (e.g., few many, much) and ‘tentative’ for PHQ-9, ‘present focus’, ‘first person singular’, ‘tentative’ and ‘3^rd^ person plural’ for GAD-7, and ‘dictionary words’, ‘auxiliary verbs’ (e.g. am, will, have), ‘first person singular’, and ‘negations’ (e.g. no, not, never) for suicidal ideation. Most of these features were among the features with the strongest correlations in the bivariate analyses (Table 3).

We then tested the reduced PLS models using only the four most robust features. The reduced models showed a larger reduction in MSE during cross-validation than the full PLS model: -13% for PHQ-9, -17% for GAD-7, and -1% for suicidal ideation (Fig 3, left column). We used the reduced PLS model with one component, as the MSE increased again when adding additional components. Fig 3 shows the beta coefficients of the regression models (middle column) and the predicted mental health scores (right column). The correlation between the predicted and observed mental scores is r = 0.44 (95%CI: 0.14, 0.67, R^2^ = 0.20) for PHQ-9, r = 0.49 (95%CI: 0.20, 0.70, R^2^ = 0.24) for GAD-7, and r = 0.36 (95%CI: 0.04, 0.61, R^2^ = 0.13) for suicidal ideation. A PLS model combining the three targets revealed that the mental health scores are correlated and can be predicted using the same linguistic features (S2 Fig in S1 File), although the reduction in RMS (-7%) is smaller than for the models predicting individual mental health scores.

### Within-subjects analysis

**Fig 4:**
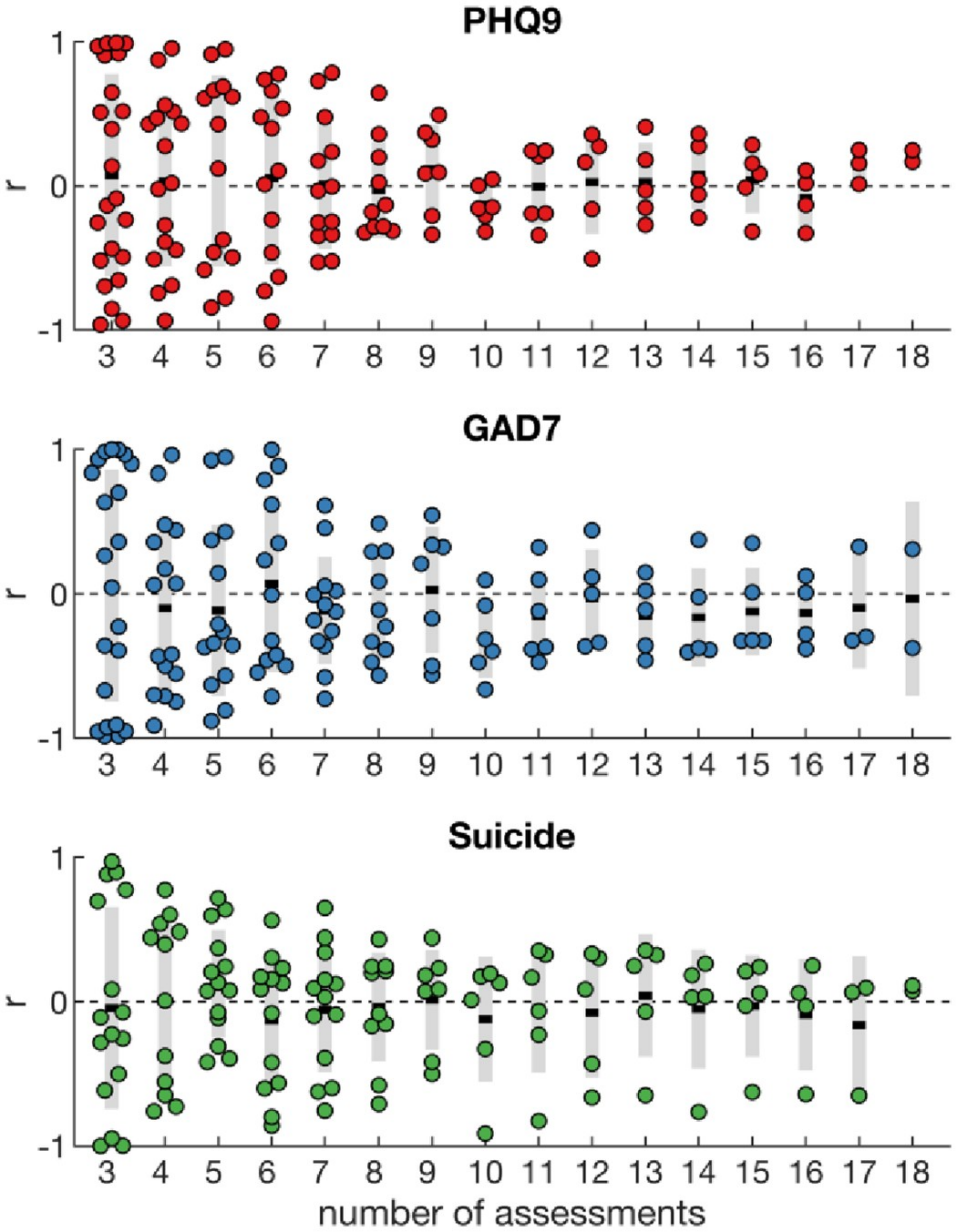
Intra-individual correlations. Group-level regression models were used to predict intra-individual changes in symptoms. The predicted mental health scores were correlated with the observed mental health scores for each participant. The coloured dots show the correlation coefficients for individual participants, the black line the group mean and the grey bars the 95% CI. The distribution of correlation coefficients was estimated for all participants having completed at least 3 to 18 assessments. The number of participants decreased with increasing number of assessments, as participants did not complete all assessments (see Fig 1B).

We used the regression models estimated from group-level data to predict within-subject variations in symptoms over time. As participants completed different numbers of assessments (Fig 1B), we tested the models multiple times for all participants having completed at least *n* assessments. The distribution of correlations coefficients across participants fluctuated around zero (Fig 4). When the correlation coefficient was estimated over a larger number of assessments the distribution become narrower; however, the 95% CI generally overlapped with zero. As such, the positive correlations observed at group level (Fig 3) were not observed for within-subject correlations. In fact, the average correlation coefficient for GAD-7 was negative when estimated over at least 10 repeated assessments.

## Discussion

This study investigated the relationship between linguistic features in blog content and individuals’ symptoms of depression, anxiety, and suicidal thinking, over a 36-week period. This study examined both group-level and individual-level correlations to test whether linguistic expression in blogs can be used to determine the mental state of individuals who use these platforms. We found mixed evidence for the hypotheses.

In the bivariate analyses of between-subjects correlations, only two linguistic features emerged as significant when controlling for the family-wise error rate: tentativeness and non-fluencies. Tentativeness, which is the degree of uncertainty reflected in text, was associated with anxiety only. This may reflect the increased worry and hesitation that characterises anxiety disorders. Non-fluencies were associated with symptoms of depression and anxiety, but not suicidal thoughts. In speech, non-fluencies relate to the various breaks and irregularities in composition, signifying pauses for thought, nervousness, or decreased alertness [41]. These have been found to be greater in depressed people’s speech [42,43] and may reflect the cognitive deficits associated with the illness. However, little is known about depression and the fluency of written text. In this study, participants with higher symptoms had fewer non-fluencies in their blogs. This is not consistent with past studies on speech patterns or other social media platforms. It may represent initial evidence of modality-specific linguistic features [18] as some social media platforms encourage individuals to communicate differently (e.g. use smaller amounts of text and short-form conventions). Variations in fluency may also be related to device use, such that bloggers using desktop or laptop devices may produce more fluent volumes of data, than those using mobile devices [44]. Our finding must also be carefully interpreted as there was a low base rate of non-fluencies in this dataset. As the LIWC tool was developed primarily from natural speech, this feature may not be as meaningful in non-spoken texts. Given the small sample and variability in data, further examination in larger datasets from various text sources and social media platforms, and devices used would help to confirm this.

Multivariate modelling revealed some similarities with the bivariate results, but also key differences as the percentage of quantifiers, dictionary words, axillary verbs, and negations used emerged as robust predictors of mental health. The differences with the bivariate analyses are partly explained by the type of correlation on which these analyses are based: we used rank-order correlation for the bivariate analyses while PLS regression finds a linear regression model. Some of the features, such as non-fluencies, were not normally distributed and hence showed significant rank-order correlation but were not among the robust features of the linear PLS model. Here we used basic regression techniques to assess the relationship between linguistic features and mental health states. More advanced machine learning techniques may be used to improve the prediction of mental health state from automated text analyses [45], but more complex models generally require large datasets to avoid overfitting [46,47]. Indeed, in the current study we found that a PLS model based on a single component had a lower prediction error than models with additional components (Fig 3).

Our findings are somewhat consistent with the previous studies that have used validated psychometric scales for depression. Similar to De Choudhury et al [13], first and third person pronouns were significant, alongside the focus on the present. These markers may convey the distancing from others and a focus on oneself that occurs in a depressive and suicidal state. The increased use of first person pronouns is also consistent with Eichstaedt et al [19] and Edwards and Holtzman’s meta-analysis [21]. This suggests that the linguistic markers of depression found in traditional forms of text, such as poetry and letters, may also be evident in blogs. In contrast to Tsugawa et al [15], negative emotion did not emerge as a significant feature of depression, anxiety, or suicidal ideation. There was also little overlap with the features found by Reece et al [14] (e.g. word count, ingestion, sadness). These contrasting findings may be related to the different social media platforms examined by these studies. There is evidence to suggest that users may adopt significantly different language styles, changing their formality and tone, across social media platforms due to differences in communication goals and contexts [48]. Different communication platforms appear to invite certain types of expressions (i.e., positive rather than negative) based on what is considered appropriate by the user community [49]. Gender and age also appear to impact self-disclosures on social media [49]. However, not all linguistic features have been measured or analysed in past studies, and the duration of our data collection was longer than most. Thus, comparing findings is challenging. This emerging and highly empirical area of research will benefit significantly from replication studies in which the same features, models, and types of data, are examined across individuals and the various platforms used.

An important strength of this study was the prospective longitudinal design. We expected that individual symptoms would fluctuate over the 36-weeks and this would be associated with a change in linguistic expression. However, this was not the case. The correlations identified at the group-level were not significant at the individual level. Thus, our findings do not support group-to-individual generalisability of linguistic markers of depression, anxiety, and suicidal thinking. In part, the lack of significant within-subjects correlations in our study may be due to missing data and the variations in blog posting, which reduced the statistical power of our analyses. There was also minimal variation in participants’ mental health scores (S1 Fig in S1 File). However, the lack of group-to-individual generalisability may also indicate that the underlying processes are indeed non-ergodic [28], that is, the relationship between linguistic features and mental health may not be equivalent across individuals and time. This represents a significant challenge to past studies and cautions the use of group-level linguistic markers for inferring individuals’ mental health status. As outlined, the relationship between linguistic features and mental health state may be specific to subgroups, such as the nature of the mental health problem, or demographics such as gender, age, and cultural identity. Patterns of linguistic expression may also differ according to the volume, type, and frequency of the collected social media data, with the language conventions, word counts, and social norms of each platform likely to influence findings [50,51].

### Limitations

While our study design had the potential to inform knowledge on the relationship between mental health symptoms and linguistic expression across time, it was hampered by low levels of data. There was significant drop-out, non-completion of the mental health assessments, and variability in the amount of blog data generated by participants. The high number of linguistic features also has the danger of inflating researcher degrees of freedom and may endanger replicability of findings [52]. As discussed, different patterns are likely to emerge from greater volumes of data, or data generated in other blog sites or social media platforms. Assessing these differences should be a pertinent focus for future research given the emerging evidence of self-disclosure biases in social media communications [53,54]. While attrition is common and seemingly unavoidable in longitudinal studies [55], the identification of markers of mental ill-health requires large amounts of individual data collected over long periods of time to effectively capture illness onset, remission and recovery. As it can be challenging to sustain human engagement in studies of this kind, and the effects of repeated measures in psychiatry is still unknown, data sharing may alleviate some of these burdens—the demand for which has been increased by open access [56]. Open access provides an opportunity to test models on datasets from multiple sources and platforms, and examine whether predictions generalise to new data [40]. Practices such as preregistration of study hypotheses and methods could also help reduce spurious correlations and will be key in identifying reliable markers of mental health state [57]. Testing predefined models to new data is likely to be the primary way for the field to advance. We have therefore shared the current dataset to help inform future studies or provide independent testing data for existing prediction models. Lastly, many of the natural language processing tools, including the LIWC, have been developed for and trained on standard language and its conventions. Such tools may underperform when applied to blogs due to the tendencies of this type of data to deviate from linguistic norms (e.g., informal language, misspellings, grammatical errors, emoticons, abbreviations, slang). Thus, our findings need to be carefully considered due to the impacts of this ‘data noise’ [58,59]. Dictionary-based analysis tools may not be sufficient to infer genuine emotional states from social media text [60] and future work should aim to account for this.

## Conclusions

Social media platforms may present an exciting opportunity for developing new tools to monitor the mental illness of individuals and populations. This study examined the associations between linguistic features in blog content and individuals’ self-reported depression, anxiety, and suicidal ideation. Several linguistic features were significantly associated with mental health scores when assessed across participants, with differences found between the bivariate and multivariate analyses. Cross-validation showed that linguistic features can predict the mental health scores of participants that were not included in the training set. When testing the multivariate regression models on longitudinal data of individual participants, no robust correlations were found between changes in linguistic features and mental health scores over time. This indicates that the model trained by group-level data could identify those with a mental illness but was not able to detect individual changes in mental health over time. This study demonstrates the importance of a prospective longitudinal study design and the use of validated psychometric scales. Future studies, utilising the advantages of open access, will need to confirm whether social media data can also be used to predict individual changes in mental health over time.

## Supporting information

S1 File (DOCX)
