# Supplementary material for: The relationship between linguistic expression in blog content and symptoms of depression, anxiety, and suicidal thoughts: A longitudinal study
Source: PLoS One. 2021 May 19;16(5):e0251787. doi: 10.1371/journal.pone.0251787 (PMC8133457; doi:10.1371/journal.pone.0251787)
Supplement: S1 File — (DOCX) [file pone.0251787.s001.docx]

**Supporting information**

**Background information on all consenting participants**

Of the 153 participants who consented to take part in the study, 127 (83%) had received a diagnosis of depression or anxiety from a medical practitioner, and 87 (57%) were taking medication for depression or anxiety. For depression scores, 22 were nil-minimal (14%), 36 mild (24%), 29 moderate (19%), 39 moderately severe (25%) and 27 severe (18%). For anxiety scores, 34 were minimal (22%), 47 mild (31%), 40 moderate (26%), 25 moderately severe (16%) and 7 severe (5%). A total of 72 (47%) reporting having had an anxiety attack in the two weeks prior, with the mean number of attacks in this period being 5.3 (SD:7.4, range: 1-60). At baseline, 81 (53%) had “thoughts that they would be better off dead, or of hurting themselves” for several days or more in the past two weeks. Also, at baseline, 68 (44%) reported both moderate to severe depression and anxiety, with depression and anxiety scores highly correlated (*r =* 0.78*, p <* .001*)*.

**Intra-individual variability**

The current study collected mental health data longitudinally and participants were asked to complete the PHQ-9 and GAD7 every two weeks for a total of 18 assessments. To assess whether participants revealed longitudinal changes in their mental health levels, we assessed variability between repeated assessments. Participants completed 5.4 assessments on average (SD: 5.2, Fig. 1B). The patterns of longitudinal change differed across participants. Several participants revealed large changes in mental health scores over time: 12 out of 38 participants had at least a 10-point differences between the lowest and highest PHQ-9 score and 7 out of 38 participants had at least a 10-point differences between the lowest and highest GAD-7 score (Fig. S1). As the Suicidal thoughts scores were based on participants’ responses to item 9 of the PHQ-9 which ranges from 0 to 3, the longitudinal change for this target was lower.


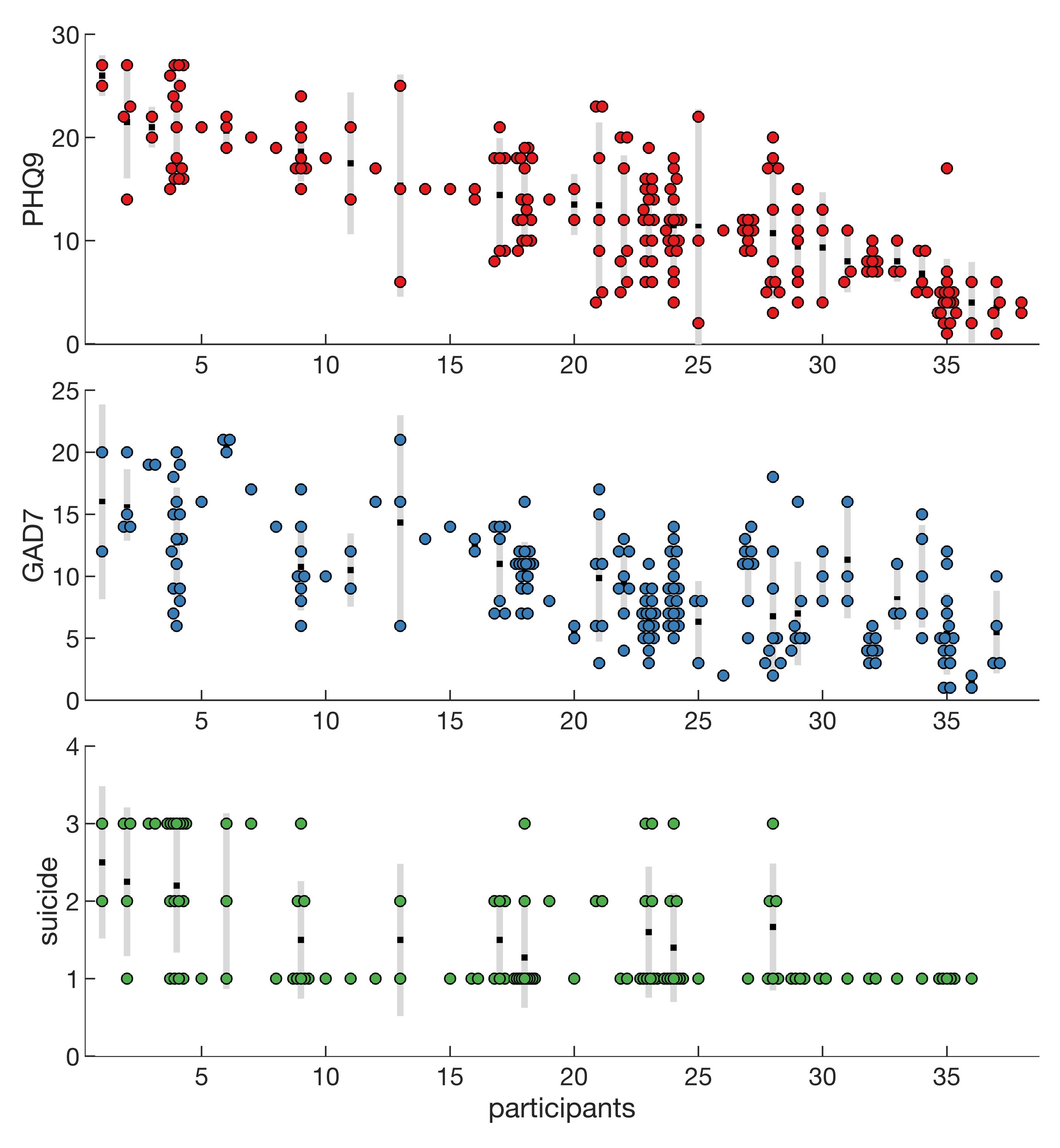


**Figure S1**. **Repeated mental health scores for individual participants (n=38).** Coloured dots show values of repeated assessments, the horizontal black line the individual mean and the grey bars the SD.

**Single PLS model to predicted combined mental health outcomes**

We assessed PLS regression models for each mental health target (PHQ-9, GAD-7 and suicide) separately. However, PLS regression also allows to predict multiple target measures using a single model. We also assessed the PLS model that predicted all three mental health scores simultaneously. Similar to the separate PLS models, the combined model showed the lowest MSE for a model with a single component. With a single component, the full model showed an increase in MSE of 0.08 (+2.6%), while the reduced model showed a reduction in MSE of 0.23 (-7.3%; Fig. S2). The reduced model explained 15% of the variance. The positive weights for the three targets reflect the positive correlations between the three mental health scores.


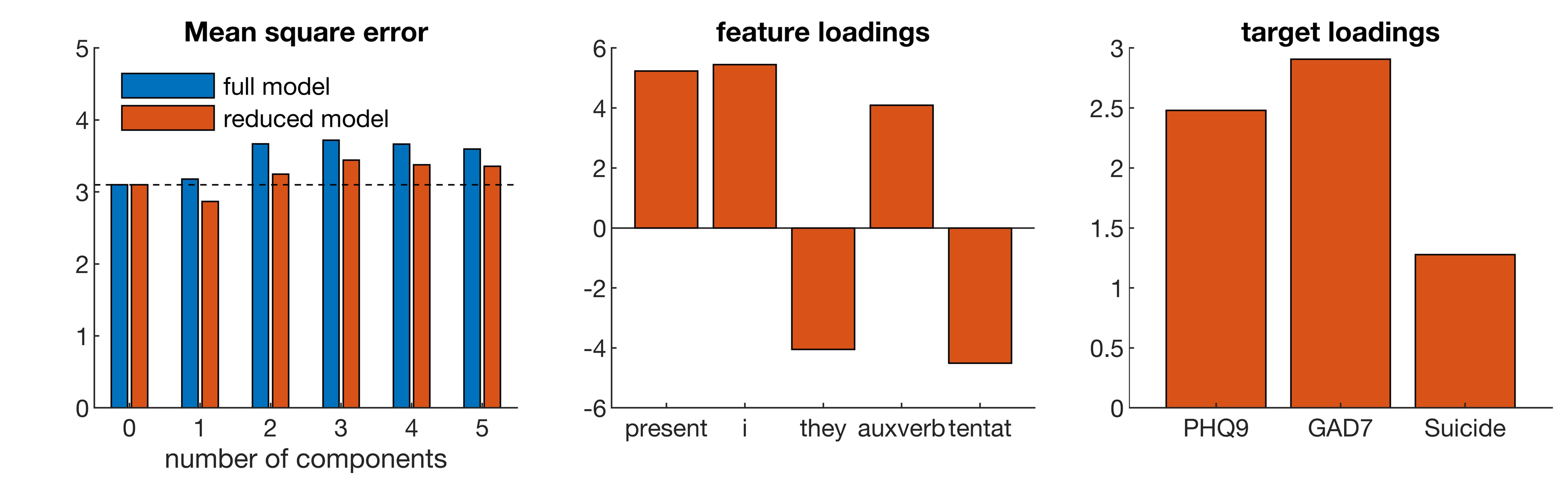


**Figure S2**. **Results of PLS regression for combined mental health outcomes.** We used 5-fold cross-validation to determine the number of PLS components. The optimal model was the model showing the lowest MSE (left panel). We tested both the full model using all 68 linguistic features and a reduced model using only the 5 most robust features. The middle panel shows the beta coefficients of the most robust features and the right column the coefficients of the three targets. Note: present=present focus, i=1^st^ person pronouns, they=3^rd^ person plural, auxverb=auxiliary verbs, and tentat=tentative.
